# Air-coupled ultrasound detection using capillary-based optical ring resonators

**DOI:** 10.1038/s41598-017-00134-7

**Published:** 2017-03-01

**Authors:** Kyu Hyun Kim, Wei Luo, Cheng Zhang, Chao Tian, L. Jay Guo, Xueding Wang, Xudong Fan

**Affiliations:** 1 Department of Biomedical Engineering, University of Michigan, 1101 Beal Ave., Ann Arbor, MI 48109 USA; 2 Department of Electrical Engineering and Computer Science, University of Michigan, 1301 Beal Ave., Ann Arbor, MI 48109 USA; 3 School of Optical and Electrical Information, Huazhong University of Science and Technology, 1037 Luoyu Road, Hongshan District, 430074 Wuhan, Hubei PR China

## Abstract

We experimentally demonstrate and theoretically analyze high Q-factor (~10^7^) capillary-based optical ring resonators for non-contact detection of air-coupled ultrasound. Noise equivalent pressures in air as low as 215 mPa/√Hz and 41 mPa/√Hz at 50 kHz and 800 kHz in air, respectively, are achieved. Furthermore, non-contact detection of air-coupled photoacoustic pulses optically generated from a 200 nm thick Chromium film is demonstrated. The interaction of an acoustic pulse and the mechanical mode of the ring resonator is also studied. Significant improvement in detection bandwidth is demonstrated by encapsulating the ring resonator in a damping medium. Our work will enable compact and sensitive ultrasound detection in many applications, such as air-coupled non-destructive ultrasound testing, photoacoustic imaging, and remote sensing. It will also provide a model system for fundamental study of the mechanical modes in the ring resonator.

## Introduction

Ultrasound detection is one of the most widely used methods to non-invasively examine internal and external structures of samples. It has been implemented in diverse fields such as medical imaging^1–3^, manufacturing^4^ and structural diagnosis^5, 6^. Ultrasound detection, however, usually requires an acoustic impedance matching layer (such as water, gel, or solid) between the sample and the ultrasound detector. Such requirement is due mainly to the high acoustic coupling loss at the sample/air (or detector/air) boundary, and the large acoustic absorption of air at ultrasonic frequencies, both of which effects significantly reduce the intensity of air-coupled pressure waves received by the ultrasound detector^7–9^. These acoustic losses make it difficult to perform ultrasound imaging when the ultrasound detector and the sample must remain contactless, such as imaging of sensitive wounds or dangerous specimens, functional ophthalmology imaging, and operating while dry environment must be maintained^5, 7, 10–12^. Therefore, non-contact ultrasound detection requires highly sensitive acoustic detectors with low noise equivalent pressure (NEP) to overcome the acoustic coupling loss and the acoustic absorption^10, 11, 13^.

For non-contact ultrasound detection, optics-based ultrasound detection techniques can be attractive alternatives to conventional ultrasound detection schemes^11, 14–26^. Unlike conventional piezoelectric or MEMs-based non-contact ultrasound detectors^10, 12, 13, 27, 28^, optical detectors do not suffer from geometry-dependent electrical noise, and are immune towards electromagnetic interference^14, 23^. Furthermore, the operating frequency is not restricted by the size (e.g., thickness and area) of the detector material^18, 24^.

Currently, there are about two different approaches in optics-based ultrasound detection techniques. First approach is remote optical detectors where the free space optical beam is used to detect the ultrasound waves, using either interferometry or beam deflection^11, 20, 25, 26^. In the interferometry configuration, back-scattered lights from the sample surface are used to detect ultrasound-induced surface displacement of the sample. In the beam deflection method, ultrasound-induced refractive index shift deflects the propagating optical beam, and then the amount of beam deflection is recorded using a quadrant photodiode. In both cases, the optical beam carries the information of the ultrasound pressure to the detector, and therefore, aforementioned absorption/propagation loss can be neglected. For this reason, remote optical detectors could be promising modalities for the non-contact ultrasound detection. Some of the previously reported NEPs were 11.4 Pa^26^, 14 Pa/√Hz^20^ and 810 mPa/√Hz^25^, with bandwidth in the order of 1–20 MHz^25, 26^. The problem with optical remote detectors, however, is relatively complex optical setup which increases the form factor. Additional problem for the interferometric detection is signal artifacts arising from the surface roughness of the sample^12^.

Another type of optics-based detection is optical resonator-based detection. In this scheme, the optical resonators detect ultrasound waves directly via photoelastic effect or physical deformation of the resonator^16–18, 22, 29–31^. While there are many different types of optical resonators for ultrasound detection (e.g., thin-film Fabry-Perot cavity^16, 17^ and akinetic contact Fabry-Perot cavity^22^), optical ring resonators are interesting because of high optical-Q factors (>10^5^) and small form factors. Over the past decade, various ring resonator ultrasound detectors have been investigated, including polymer ring resonators on a chip^19, 32–34^, silicon/silicon nitride ring resonators on a thin membrane^35, 36^, and fused silica microresonators^31, 37^. Due to the high optical Q-factors of the whispering gallery mode (WGM), the ring resonator can achieve high pressure sensitivity with reported NEPs with 0.4 Pa^35^, 574 μPa/√Hz^32^, 2.5 mPa/√Hz^38^ and 6 mPa/√Hz^19^. The reported bandwidths are on the order of 1–350 MHz^19, 24, 32, 39^. With lower reported NEPs than remote optical detectors, ring resonators have a small form factor (typical ring diameter: 50–200 μm). Such a small form factor is advantageous in building an array of detectors. Furthermore, since the resonators directly detect ultrasound waves, the surface roughness of the sample causes less problem than interferometric detectors discussed previously.

However, to date, all ring resonator ultrasound detection is carried out by utilizing coupling media such as water. Here, we developed an air-coupled ultrasound detector using high Q-factor (>10^7^) ring resonators based on fused silica capillaries. Ultrasound at 50 kHz and 800 kHz were detected with an NEP of 215 mPa/√Hz and 41 mPa/√Hz, respectively. Furthermore, the responses of the ring resonator were compared with commercially available detectors. Finally, air-coupled detection of PA ultrasound pulses generated from a 200 nm Chromium (Cr) film by a 6 ns pulsed laser was achieved.

## Theory

In the ring resonator, light is coupled into a WGM and circulates along the resonator circumference. Upon being impinged on by pressure waves, the WGM undergoes a spectral shift due to the changes in the refractive index (photoelastic effect) and the shape (deformation) of the ring resonator. This spectral shift can be detected directly in the spectral domain or indirectly by monitoring the light transmission intensity^29, 40^.

The resonant wavelength *λ* of the WGM is governed by:

1 $$nl=m\lambda $$

where *n* and *l* are the effective refractive index and the circumference of the ring resonator, respectively. *m* is the azimuthal mode number. The WGM undergoes a spectral shift upon being impinged on by a pressure wave due to the photoelastic effect (*Δn*) and the geometric deformation (*Δl*), which can be expressed as:

2 $$\frac{{\rm{\Delta }}\lambda }{\lambda }=\frac{{\rm{\Delta }}n}{n}+\frac{{\rm{\Delta }}l}{l}$$

In our experiments, the capillary had a bottle-like geometry in order to confine the WGM in the longitudinal direction. Therefore, it can be locally (near its equator) approximated as a hollow microsphere. We further assume that the sphere size is much smaller than the ultrasound wavelength in air. Consequently, *Δn*/*n* and *Δl*/*l* due to the pressure difference can be derived as^40–42^:

3 $$\begin{array}{rcl}\frac{{\rm{\Delta }}n}{n} & = & \frac{(3{p}_{i}{b}^{3}-{p}_{o}{b}^{3}-2{p}_{o}{a}^{3}){C}_{1}+(3{p}_{i}{b}^{3}+{p}_{o}{b}^{3}-4{p}_{o}{a}^{3}){C}_{2}}{2n({a}^{3}-{b}^{3})}\\  & \approx  & -\frac{3\chi ({p}_{o}-{p}_{i})({C}_{1}+{C}_{2})}{2n}\\  & \approx  & -\frac{3\chi ({p}_{o}-{p}_{i})({C}_{1}+{C}_{2})}{2n}\end{array}$$

4 $$\begin{array}{rcl}\frac{{\rm{\Delta }}l}{l}=\frac{{\rm{\Delta }}r}{r} & = & \frac{(4G+3K){p}_{i}{b}^{3}-4G{p}_{o}{a}^{3}-3K{p}_{o}{b}^{3}}{12GK({a}^{3}-{b}^{3})}\\  & \approx  & \frac{-\chi ({p}_{o}-{p}_{i})(4G+3K)}{12GK}\end{array}$$

where *p*_*i*_ and *p*_*o*_ are the inner and outer pressure, respectively. *C*_*1*_ and *C*_*2*_ are stress-optic tensor constants, *G* is the shear modulus and *K* is the bulk modulus. *G* and *K* for silica are 31.2 GPa and 36.7 GPa, respectively. *a* and *b* are the outer and inner radii, and *χ* is the geometrical parameter, *a* ^*3*^/(*a* ^*3*^ − *b* ^*3*^). For silica, *C*_*1*_ is −3.03 × 10^−12^ m^2^/N at 1550 nm^43^. Unfortunately, *C*_*2*_ at 1550 nm is unavailable. By comparing with the ratio between *C*_*1*_ values in the visible spectrum and 1550 nm, *C*_*2*_ in 1550 nm wavelength can be approximated as −0.47 × 10^−12^ m^2^/N^44^. For a ring resonator with *a* = 65 μm and *b* = 55 μm, *Δλ*/*λ* is calculated to be −3.41 × 10^−11^ per Pascal. Based on Eqs (3) and (4), pressure sensitivity can be enhanced by using thinner-walled ring resonators with larger radii.

## Methods

### Experimental Setup

**Figure 1:**
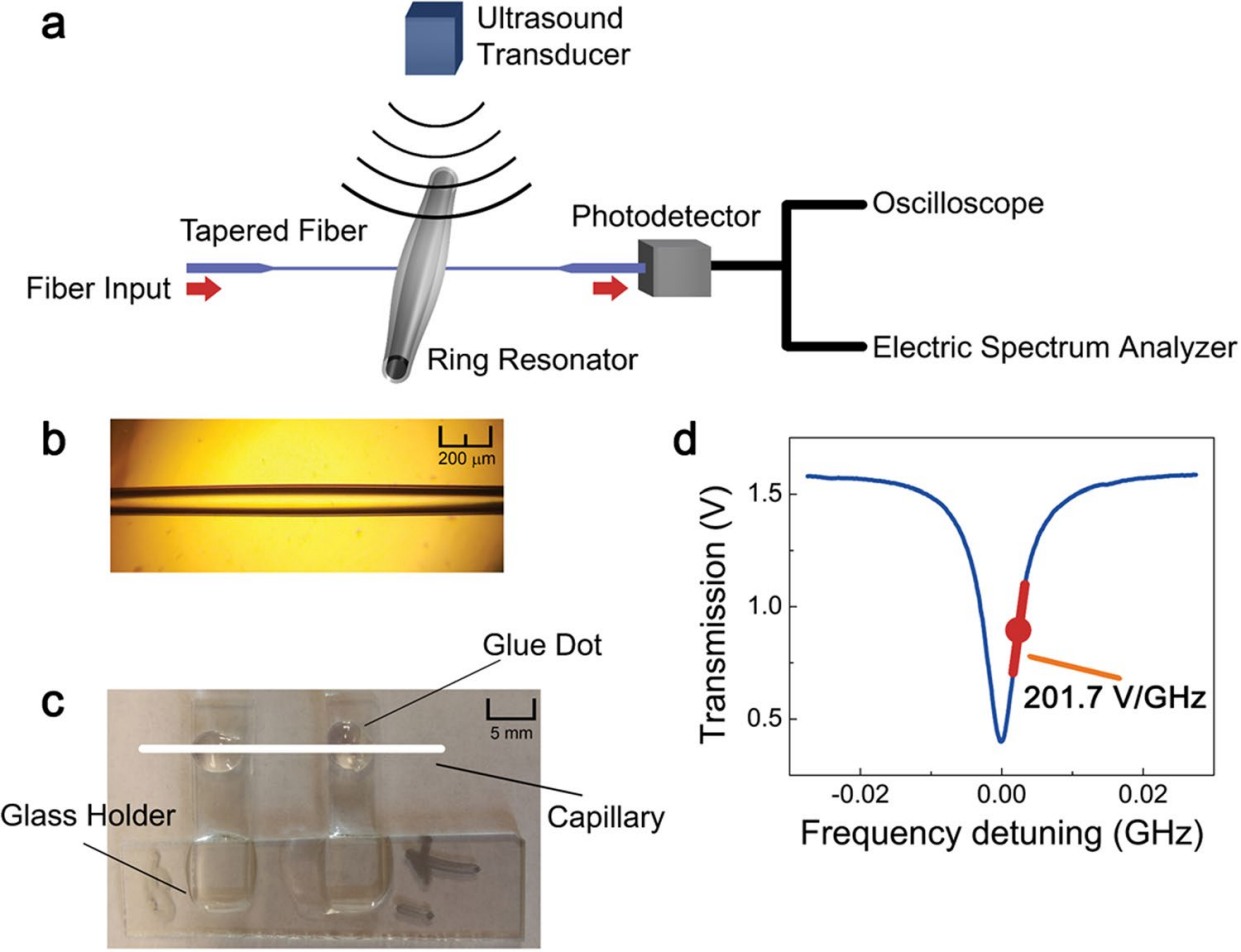
(**a**) Schematic of the experimental setup for pressure wave detection using a capillary-based ring resonator. An optical fiber taper was placed in contact with the capillary to couple the laser light around 1550 nm from a tunable laser into the WGM of the ring resonator. The transmitted light was detected by a photodetector (either DC-coupled or AC-coupled) and analyzed by an oscilloscope and a spectrum analyzer. An ultrasound transducer (800 kHz) was placed 2 cm above the resonator, aiming at the center of the resonator. (**b**) Photograph of the capillary-based bottle-shaped ring resonator. (**c**) Photograph showing the capillary-based ring resonator placed on a holder and secured by two glue dots. The distance between the two glue dots could vary, but did not affect the pressure measurement. The minimum distance tested was 3 mm. The white line indicates the position of the capillary. (**d**) Transmission signal shows that the Q-factor of the ring resonator (65 μm in outer radius and 10 μm in wall thickness at the most-bulged part) was 3.5 × 10^7^. The red dot shows the spectral position where the laser was fixed. Slight WGM spectral shift resulting from a pressure wave causes the transmission intensity to change.

Figure 1a illustrates the experimental setup for air-coupled ultrasound detection using a capillary-based ring resonator. 1550 nm light from a tunable diode laser (New Focus 6328) was sent through an optical fiber (SMF-28e). The optical fiber consists of a tapered region where the light can be evanescently coupled into the WGM mode of the resonator^45^. The tapered region was prepared by melting the optical fiber using a hydrogen torch while pulling the fiber sideways. Typical diameter of tapered region was 1–2 μm. The tapered region was placed in contact with the resonator to couple the light from the tunable diode laser. Optical power of ~1 mW was coupled into the resonator. Upon being impinged on by ultrasound, the WGM of the resonator undergoes a spectral shift due to the change in the refractive index and ring perimeter as described above. The circulating light that experienced the spectral shift couples back to the tapered fiber, and then travels to the photodetector which is placed at the end of the tapered fiber. The amount of resonance wavelength shift due to the pressure was detected as a transmission intensity change at the photodetector^23, 33, 41^. The detail about the relationship between the optical transmission intensity and the resonance wavelength shift within the WGM ring resonator is well described in the review article by Zhang *et al.* ^19^. The transmission was measured by either a DC-coupled (New Focus 1811, DC – 125 MHz bandwidth) or an AC-coupled (New Focus 1611, 30 kHz – 1 GHz bandwidth) photodetector, and analyzed by an oscilloscope (Tektronix DPO 3014) and a spectrum analyzer (Agilent N9010A). Fiber-coupled optical attenuators were used to operate photodetectors near the saturation threshold. Tektronix DPO 3014 has a bandwidth of 100 MHz and the sample rate was set at 500 MS/s, 1.25 GS/s or 2.5 GS/s, depending on the experiments. Spectrum analyzer was used to monitor the spectrum in real-time, but not to obtain data. An 800 kHz air-coupled unfocused ultrasound transducer (Japan Probe 0.9K14x20N-RX) was placed 2 cm above the resonator, aiming at the center of the resonator. The orientation of the transducer relative to the longitudinal axis of the capillary did not produce any noticeable difference during this experiment.

### Device Preparation and Fabrication

The ring resonator was fabricated by stretching a fused silica capillary under amplitude-modulated CO_2_ laser illumination, which generates bottle-like curvatures along the capillary longitudinal axis^45, 46^ (Fig. 1b). The capillary had a typical outer radius of 65–90 μm and a wall thickness of 7–15 μm, which can be customized during the pulling process. The longitudinal size (length) of the resonator was about 1 mm. The capillary was subsequently secured on a U-shaped glass holder via two glue dots (Fig. 1c), whose distance could vary but did not affect the pressure measurement. The detailed description of how taper is used to couple the light into a capillary resonator is well documented in the article by Han *et al.* ^45^. Figure 1d plots the transmission spectrum of the ring resonator (65 μm in outer radius and 10 μm in wall thickness at the most-bulged part) by scanning the tunable laser frequency, showing a Q-factor of 3.5 × 10^7^, consistent with that in our previous studies^46, 47^. During the ultrasound measurement, the laser frequency was fixed at the quadrature point (the red dot in Fig. 1d) to achieve the maximal pressure sensitivity.

## Results

### Validation of the Theoretical Analysis

**Figure 2:**
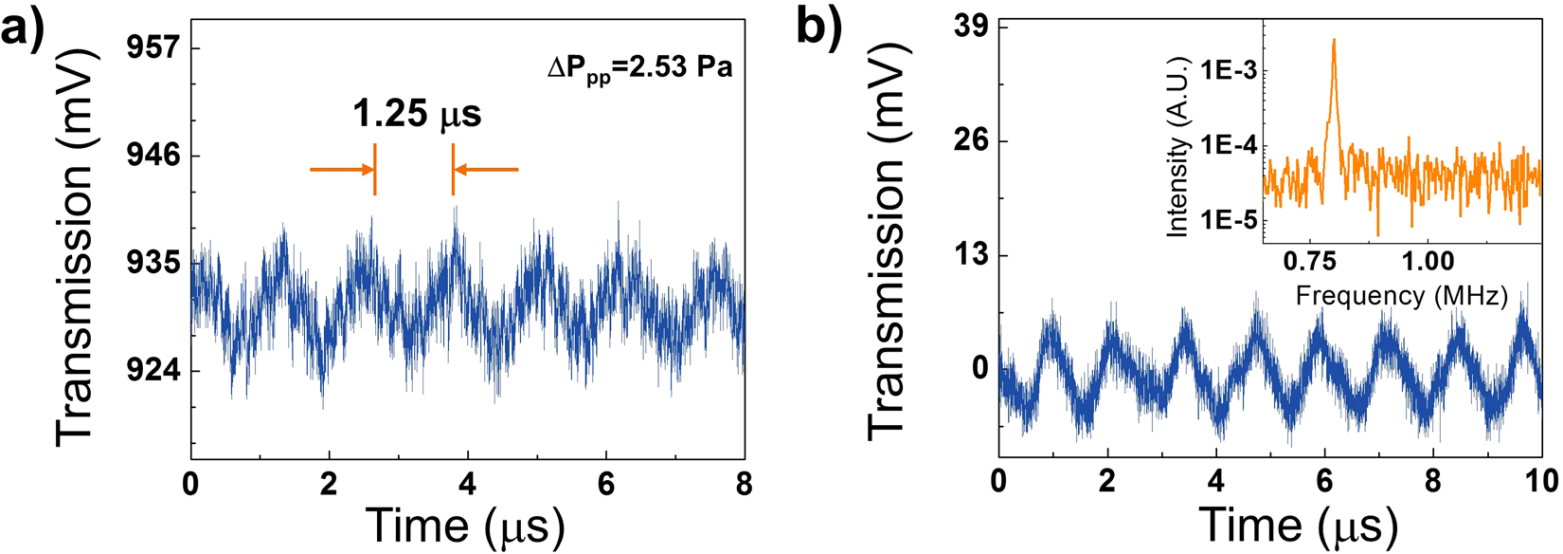
(**a**) Temporal trace detected by a DC-coupled photodetector connected to an oscilloscope. The peak-to-peak pressure at the resonator was 2.53 Pa (see Fig. S2b for the pressure calibration curve). 1.25 μs period corresponds to the 800 kHz pressure wave from the air-coupled ultrasound transducer. (**b**) Temporal trace detected by an AC-coupled photodetector. The temporal response of the ring resonator (*a* = 90 μm) for 10.3 Pa (peak-to-peak) incident pressure wave. Inset: Spectral response of the ring resonator obtained using fast Fourier transform.

We first experimentally validate the theoretical analysis presented above. The resonator used here was the same as the one shown in Fig. 1d. Continuous ultrasound of 800 kHz was generated by an air-coupled ultrasound transducer (Japan Probe 0.9K14x20N-RX) placed 2 cm above the resonator. To characterize the ring resonator pressure sensitivity, we first used the DC-coupled photodetector. Figure 2a shows the temporal trace of the transmission light detected by a DC-coupled photodetector. The peak-to-peak pressure at the resonator was 2.53 Pa (see the Supplementary Information for details of the transducer pressure calibration). An oscillation with a period of 1.25 μs that corresponds to 800 kHz is clearly observed. The peak-to-peak voltage change is approximately 15 mV. Based on the slope of 201.69 V/GHz from Fig. 1d, the sensitivity of the WGM spectral shift to the pressure is estimated to be approximately 30 kHz/Pa, which translates to a fractional resonance spectral shift of 1.5 × 10^−10^ per Pascal at 1550 nm. This result is consistent with previously reported pressure sensitivity of hollow fused silica ring resonators under static pressure^42^, after adjusting for the diameter and thickness of our resonator.

It is also comparable to the theoretical value of −3.41 × 10^−11^ per Pa discussed in the “Detection principle Theory” above. The difference between the theoretical analysis and the experimental measurement may be attributable to the spherical approximation of the ring resonator and the assumption that the ring resonator size (65 μm in radius) is much smaller than the ultrasound wavelength (430 μm for 800 kHz in air).

**Figure 3:**
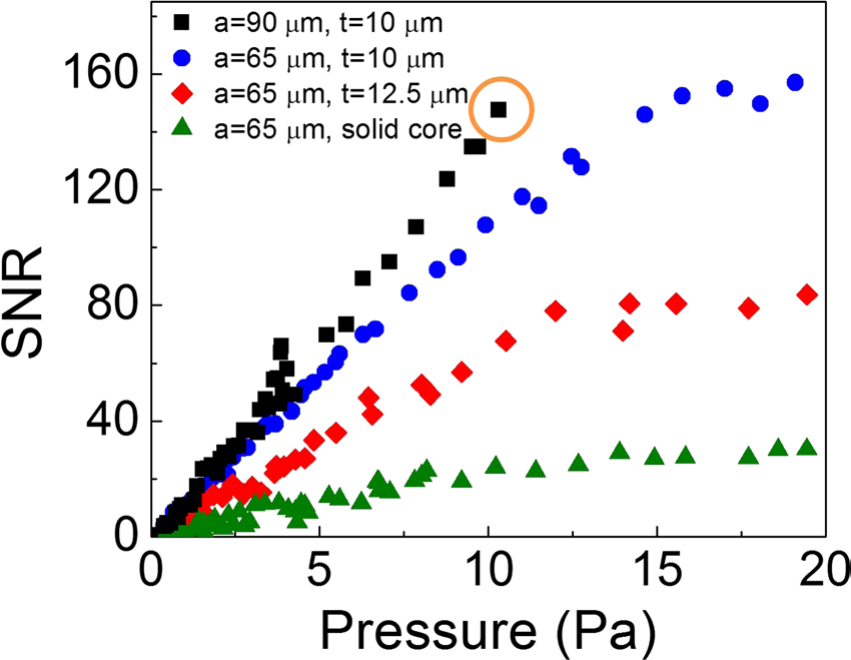
SNR vs. various 800 kHz ultrasound peak-to-peak pressures for three capillary-based ring resonators and a solid-core ring resonator. The physical dimensions of the capillaries are given in the legend of the plot. *a* is the outer diameter; *t* is the wall thickness. The slope in the linear region represents the ring resonator pressure sensitivity. The results show that larger resonators with thinner wall have higher pressure sensitivity, as predicted by Eqs (3) and (4). The temporal response for the data point in the orange circle is given in Fig. 2b.

We then investigated the sensitivity dependence on the ring resonator diameter and wall thickness. For those studies and the subsequent experiments, we chose to use the AC-coupled photodetector to remove the DC-noise. An exemplary temporal response from a resonator using an AC-coupled photodetector is demonstrated at Fig. 2b. The responses of the ring resonators to various impinging pressures are shown in Fig. 3. SNR was measured at the spectral domain by calculating the ratio of peak signal to the noise level. For the smaller resonator (*a* = 65 μm, blue dots), at relatively low pressures, the response increases linearly with the increased pressure, as seen in Fig. 3 (blue circles). Beyond 15 Pa (peak-to-peak), the ring resonator response starts to level off. As suggested in Eqs (3) and (4), the thickness of the ring resonator affects its pressure sensitivity. Experimentally, the ring resonators with the same outer radius but different thicknesses were tested using the same experimental setup as shown in Fig. 3, showing that the thinner-walled capillary is more sensitive to pressure than the thicker-walled counterparts. For a larger resonator (*a* = 90 μm, black squares), the pressure sensitivity is higher than the smaller resonator, as seen in Fig. 3 (black squares).

### Device Performance Evaluation

After verifying the fundamental model of the ring resonator in response to ultrasound waves, we measured the ring resonator’s NEPs at two different ultrasound frequencies, 50 kHz and 800 kHz. At the same time, ring resonator’s performance was compared with a commercial calibrated microphone (G.R.A.S. 46BE with a preamplifier) designed for 4 Hz–100 kHz ultrasound, and an air-coupled unfocused ultrasound transducer (Japan Probe 0.9K14x20N-RX) designed for 800 kHz ultrasound (Note: note that another piece of Japan Probe transducer was also used to generate 800 kHz ultrasound in later of our experiments). For this experiment, optical signals were measured using a New Focus 1611 photodetector.

For the 50 kHz ultrasound measurement, we used a ring resonator with the outer radius of 60 μm and the wall thickness of 10 μm. The detectors (ring resonator and Japan Probe transducer) were placed 23 cm away from an air-coupled 50 kHz ultrasound transducer (AirMar AR 50), which generated a peak pressure of 7 Pa at the detector’s location, as measured by the calibrated microphone. During the experiment, the 800 kHz transducer was placed right behind the ring resonator. Due to the small size of the ring resonator, the receiving face of the 800 kHz transducer was not obstructed by the resonator, thus allowing the response of the ring resonator and 800 kHz transducer to be taken simultaneously. Note that since the attenuation of 50 kHz ultrasound in air is about 1.66 dB/mm^9^, the pressure difference caused by the small gap (a few millimeters) between the 800 kHz transducer and the ring resonator can be ignored. The response of the calibrated microphone was taken using the same experimental setup in a separate measurement, since the 800 kHz transducer and the microphone could not be placed together. The microphone was placed ~23 cm away from the air-coupled 50 kHz ultrasound transducer. The peak pressure of 8 Pa was detected for the microphone experiment.

**Figure 4:**
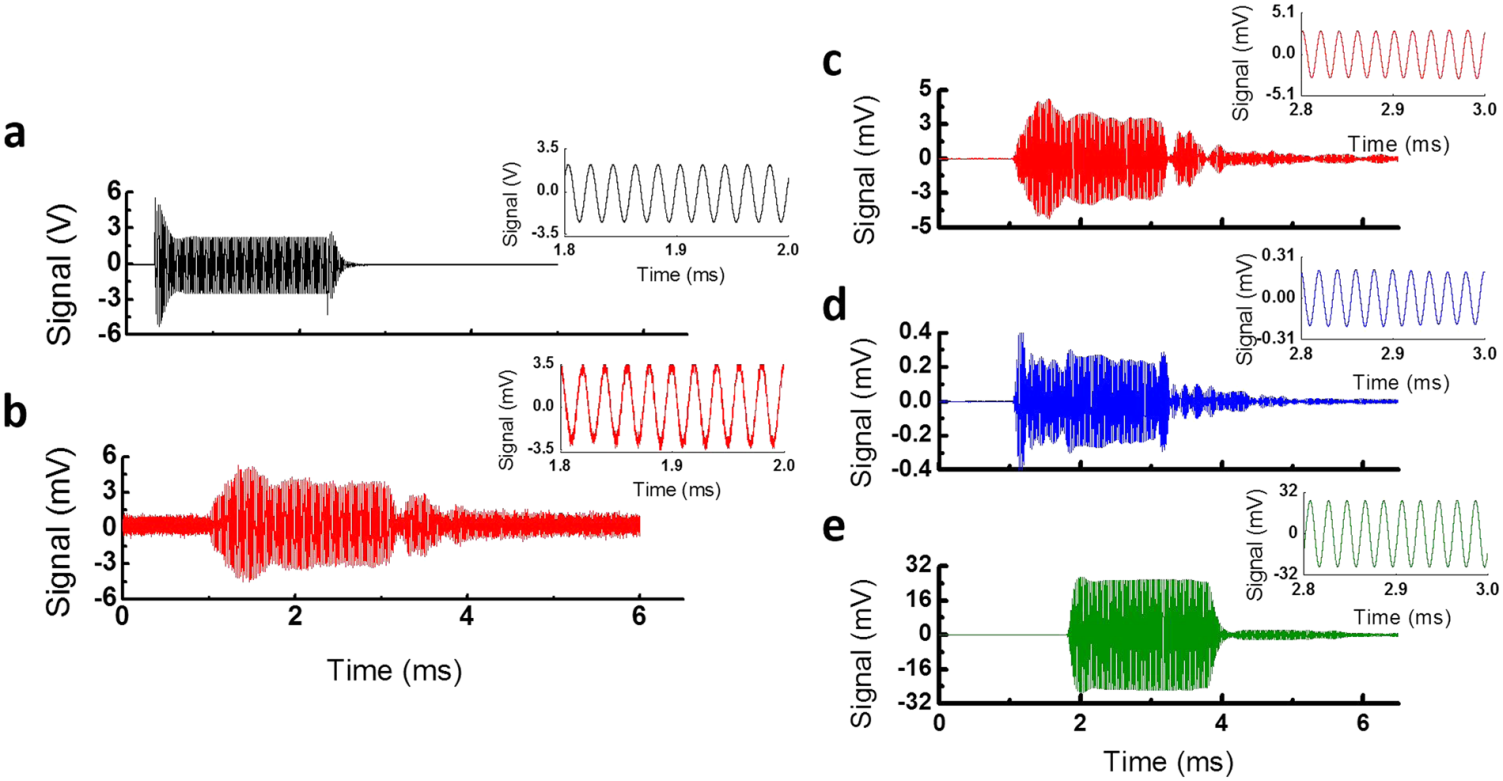
Temporal response of different ultrasound detectors to a 50 kHz air-coupled ultrasound transducer (AirMar AR 50). Transmit ultrasound burst of 100 cycles was generated. All data were averaged 256 times. Response data in (**c**,**d** and **e**) are post-filtered with a bandpass filter with a *f*_*0*_ = 50 kHz and *Δf* = 20 kHz. Insets show sinusoidal temporal signals for each detector. (**a**) Driving voltage input to the 50 kHz transmitting transducer. Sinusoidal burst output with a center frequency of 50 kHz was sent to the transducer. (**b**) Detected transmission signal from the ring resonator. (**c**) Post-filtered transmission signal from the ring resonator with a bandpass filter. (**d**) Post-filtered signal from the 800 kHz ultrasound transducer. (**e**) Post-filtered signal from the microphone.

Figure 4b shows the temporal response of the ring resonator towards 50 kHz ultrasound. This response data was obtained by averaging over 256 samples. The 7 Pa incident ultrasound wave induced 5 mV peak signal. The SNR of the ring resonator detector was calculated from the ratio of peak signal to the root-mean-square (RMS) of the noise level before the ultrasound signal (*e.g*., baseline signal before ~1.2 ms in Fig. 4b). By taking the averaging factor into account, we obtain the unaveraged SNR of 1.25 for the detected signal. From the SNR and the detected bandwidth of the incident ultrasound signal, we calculated the NEP to be 215 mPa/√Hz at 50 kHz.

Now, we compare our detector’s performance to commercial detectors. The results of the experiments are given in Table 1. Figure 4c,d and e) show temporal responses of the detectors towards 50 kHz air-coupled ultrasound. Again, response data were averaged 256 times. The data were post-filtered with a band-pass filter with a center frequency (*f*_*0*_) of 50 kHz and a full width at half maximum (FWHM, *Δf*) of 20 kHz to remove frequency noise from other frequency range. The bandwidth of the filter was large enough to capture the incident ultrasound signal. Bandpass filtering ensures that we can fairly compare all devices at the 50 kHz (i.e., microphone has a preamplifier whereas others do not). The SNR of each detector was determined using the RMS noise before the ultrasound signal. SNRs of each detector are presented in Table 1. In the table, values of SNR have taken into the averaging factor account by dividing them by √256. The SNR of the ring resonator after filtering is about 28. The microphone had about 10*X* better sensitivity, as it is designed specifically for the detection of the ultrasound below 100 kHz. In contrast, when compared with the 800 kHz transducer, which is designed to operate at 800 kHz, the ring resonator demonstrated about 30% better SNR.

**Table 1:** SNR of Ultrasound Detectors at 50 kHz and 800 kHz.

Detector	50 kHz	800 kHz
	SNR	SNR
**Ring Resonator**	28	7
**800 kHz Transducer**	21	25
**Microphone**	279	1 (Distance: few mm)

**Figure 5:**
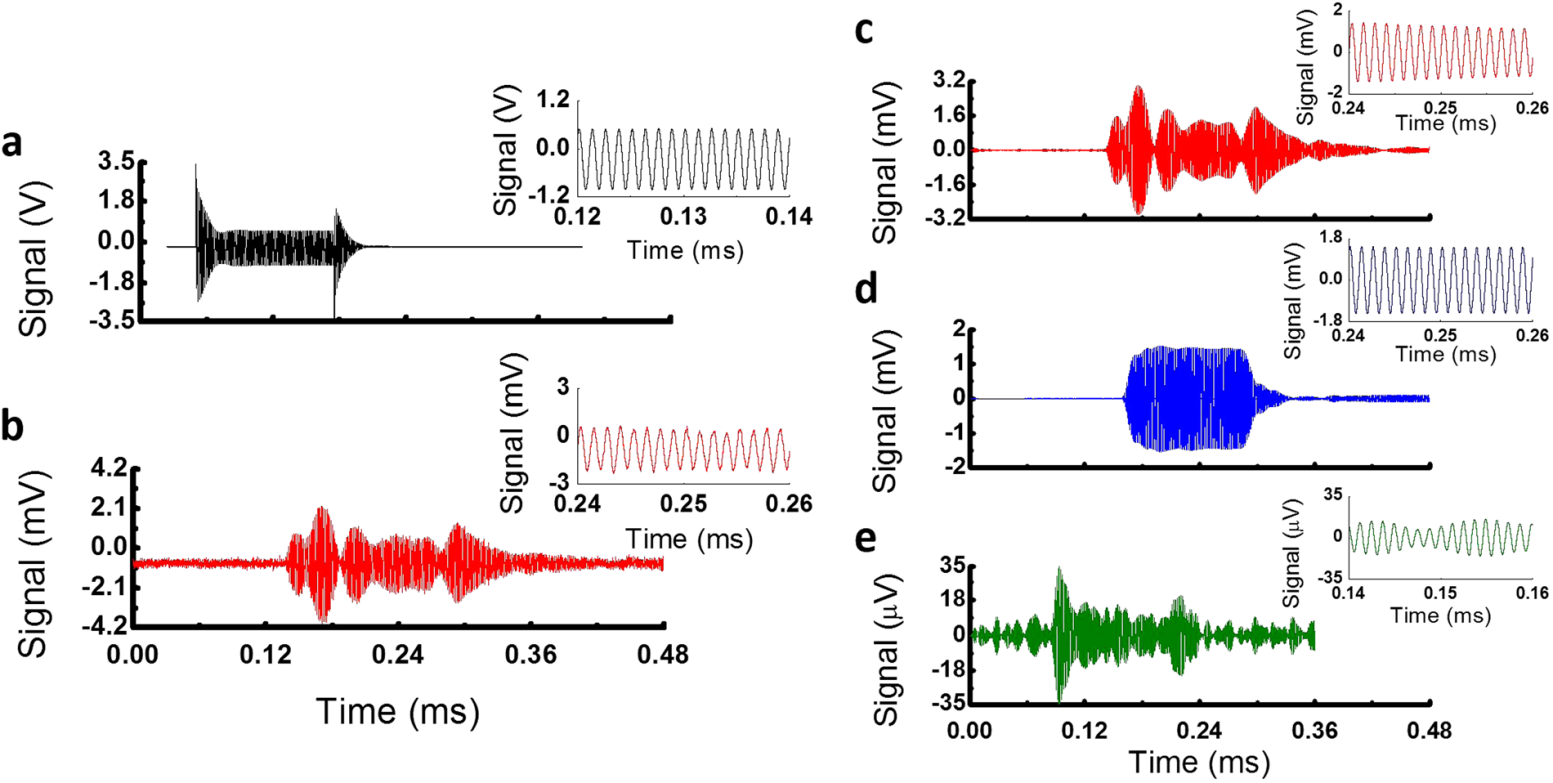
Temporal response of different ultrasound detectors to an 800 kHz air-coupled ultrasound transducer (Japan Probe 0.9K14x20N-RX). Transmit ultrasound burst of 100 cycles was generated. All data were averaged 512 times. Response data in (**c**,**d** and **e**) are post-filtered with a bandpass filter with *f*_*0*_ = 800 kHz and *Δf* = 200 kHz. Inset shows sinusoidal temporal signals for each detector. (**a**) Driving voltage input to the 800 kHz ultrasound transmitting transducer. Sinusoidal burst output with center frequency of 800 kHz was sent to the transmitter. (**b**) Detected transmission signal from the ring resonator. (**c**) Post-filtered response of ring resonator. (**d**) Post-filtered signal from the 800 kHz receiver. (**e**) Post-filtered signal from the microphone.

We now measured the ring resonator’s response to 800 kHz. An 800 kHz ultrasound transducer (Japan Probe 0.9K14x20N-RX) operating at the transmitting mode was used as the ultrasound source. As before, the ring resonator and the 800 kHz transducer (Japan Probe 0.9K14x20N-RX) operating at the receiving mode were placed together ~3 cm from the ultrasound source. Incident peak pressures were 5.47 Pa for ring resonator and 4.82 Pa for 800 kHz receiving transducer (Rx), as estimated from the response of the 800 kHz transducer (see Supplementary Information for the calibration procedure) and by taking into account the large attenuation of 800 kHz ultrasound in air (104 dB/m^9^)). For the microphone, the incident peak pressure was ~10 Pa. Figure 5b shows the temporal response of the ring resonator towards 800 kHz ultrasound. This response data was obtained by averaging over 512 samples. From the unaveraged SNR of 1.4, we arrived at the NEP to be 41 mPa/√Hz at 800 kHz.

We now compare the same set of detectors towards 800 kHz ultrasound waves. The results of the experiments are given in Table 1. Figure 5c,d and e) show temporal responses of detectors toward 800 kHz transmitted ultrasound. All data were averaged 512 times and post-filtered with a band-pass filter with *f*_*0*_ = 800 kHz and *Δf* = 200 kHz. Detected 800 kHz signal lied inside the bandpass filter. When compared with the 800 kHz transducer, the ring resonator demonstrated about 3.5 times lower SNR. However, the ring resonator was about 6 times more sensitive than the microphone. For further comparison, we also used an imprinted polymer ring resonator based ultrasound detector developed in our group^19^ to replace the current capillary based one and no response was observed for the same 800 kHz ultrasound in air.

### Photoacoustic Detection

**Figure 6:**
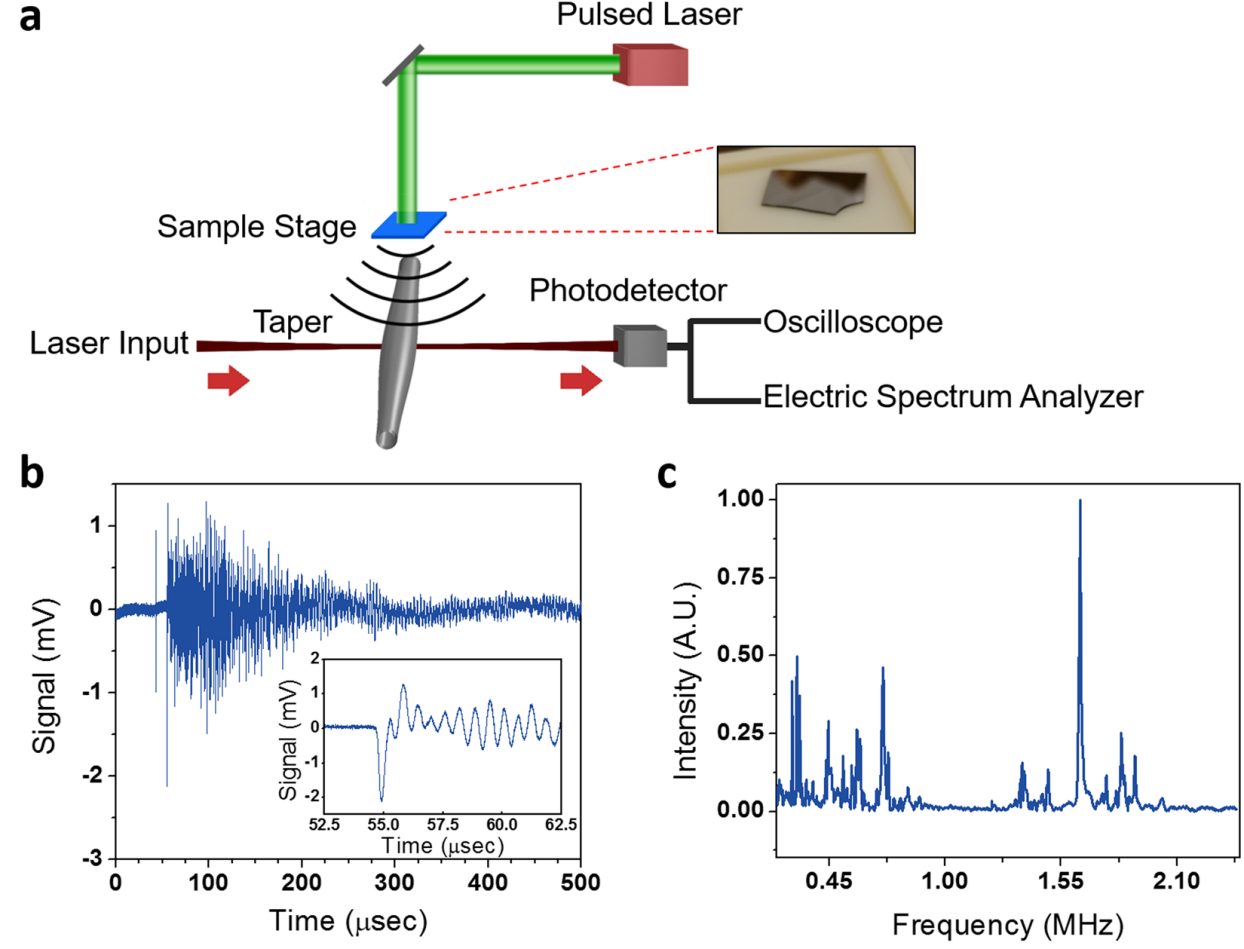
(**a**) Schematic of the PA detection setup. A 6 ns pulsed laser at 532 nm was impinged onto a 200 nm thick Cr film deposited on a silica wafer (see Inset) to generate PA signal. (**b**) Temporal air-coupled PA signal detected by a ring resonator (*a* = 80 μm, *t* = 11 μm). The data were averaged 64 times. Inset: The “ringing” response can be seen after the initial peak signal. (**c**) Frequency response of the ring resonator towards PA signal. Frequency dependent absorption in air has been taken into account. A strong peak at 1.65 MHz and small side peaks were visible.

In Fig. 6, we studied the ring resonator’s performance in detecting air-coupled PA photoacoustic (PA) signals. In this experiment, PA signal was generated by illuminating a 6 ns pulsed laser (Continuum Surelite I-20, Repetition rate: 20 Hz) onto a 200 nm thick Cr film deposited on a silica wafer (Fig. 6a)^24, 48, 49^. The thickness of the silica wafer was 500 μm. The silica wafer with Cr film was then diced into a smaller bar. The length of the bar was ~2 cm, while the width was ~6 mm (Fig. 6a, Inset). The bar was then placed ~4 mm above the resonator. The resonator used in this experiment had an outer radius of 80 μm and a thickness of 11 μm. Figure 6b plots the resonator’s temporal response to a PA pulse generated by the Cr film placed 4 mm away, illuminated by 850 μJ/pulse laser pulses. The beam diameter at the sample was 2.5 mm. The generated PA signal had a peak pressure of ~1 Pa, as measured by the calibrated microphone (G.R.A.S. 46 BE). The first peak around 40 μs was an electrical artifact coming from the laser trigger signal. This peak was also present even when the laser shutter was closed. The peak around 55 μs was the signal from the PA signals (Fig. 6b inset). Considering the RMS noise of 28 μV, which was measured with the laser shutter closed, the SNR of 10 (the averaging factor has been taken into account) was obtained.

Assuming that blood is an optical absorber, with ANSI safety limit laser fluence *F* = 20 mJ · cm^−2^ and optical absorption coefficient *μ*_*a*_ = 234 cm^−1^ at 532 nm, initial photoacoustic pressure generated in blood can be calculated to be *P*_*0*_ = *Γμ*_*a*_ *F* = 0.2 · 4.68 MPa = 936 kPa, where *Γ* = 0.2 is Grüneisen parameter for blood^50^. Since the acoustic transmission coefficient of water/air interface is −65.4 dB^13^, the photoaoustic pressure coupled into air is estimated to be ~520 Pa. Assuming that the laser spot size on the sample is 10 µm, and the ring resonator is 5 mm away from the photoacoustic source, the final acoustic pressure reaching the sensor is expected to be ~520 mPa. Since our detector can detect PA signal with 1 Pa peak signal with SNR of 10, we could theoretically detect air-coupled PA signal of blood.

It should be noted that the time response exhibits the “ringing” oscillation, implying that device has a certain resonance frequency. Since the capillary based ring resonator is known to be a good mechanical oscillator with a high mechanical Q-factor^46, 51^, broadband PA pulses may excite mechanical modes of the ring resonator. In this experiment, the mechanical oscillation belongs primarily to the wineglass mode^51, 52^ due to the cylindrical symmetry of the device and the direction of the impinging acoustic pulses. In Fig. 6c, frequency response of the ring resonator is presented based on the results in Fig. 6b. The strong peak at 1.65 MHz suggests that PA pulse couples into a mechanical mode. Using the finite element calculation tool (COMSOL Multiphysics), we confirmed that 1.65 MHz corresponded to the wineglass mode^51, 52^ as described in Supplementary Information S3 and Fig. S4. The bandwidth of this mode is only 10 kHz.

**Figure 7:**
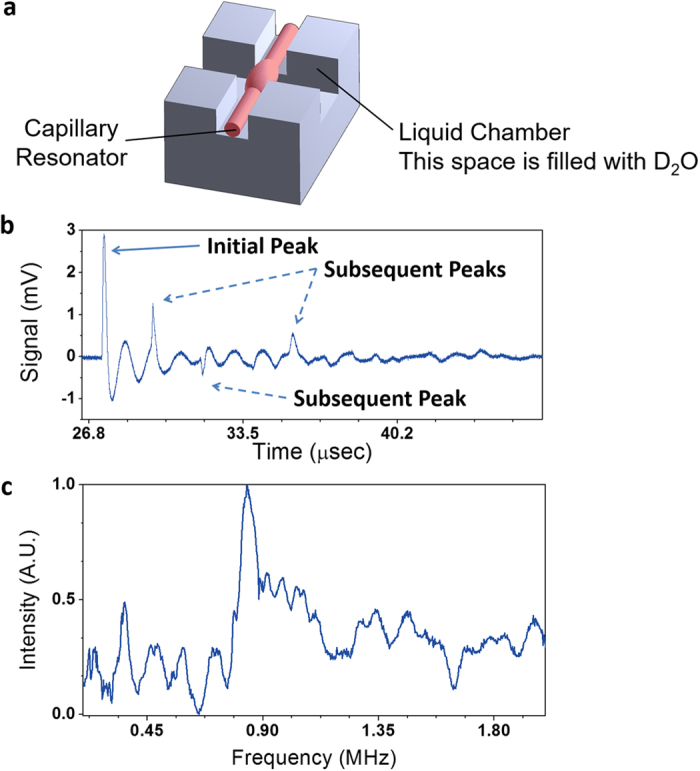
(**a**) Schematic of the chamber to immerse the ring resonator (colored in red) into D_2_O. The optical taper was placed on top of the ring resonator. The air-gap between the Cr film and the D_2_O chamber was ~1.4 mm. The distance between the top surface of D_2_O and the ring resonator was 2.0 mm. (**b**) Air-coupled PA signal detected by the ring resonator (*a* = 65 μm, *t* = 7.5 μm), immersed in D_2_O. The data were averaged 64 times. The PA peak around 27 μs was the signal from the PA pulse. The dampened “ringing” response can be seen after the initial peak signal. (**c**) Frequency response of the ring resonator towards PA pulse. A broadened peak at 840 kHz with a FWHM of 200 kHz can be obtained with D_2_O encapsulation.

In PA detection, however, such narrow frequency response is not desired^19, 53^. Given that the Cr film is a broadband PA source^48^, the PA detector’s temporal response should be sharp if it is of broadband^19, 32^. While designing an optomechanical resonator with a broadband response can be challenging, one simple solution would be encapsulation of the ring resonator using a polymer or liquid cladding to dampen the oscillation. In Fig. 7a, we inserted a resonator (*a* = 85 μm, *t* = 7.5 μm) into a chamber filled with D_2_O (heavy water)^54^. The reason to use D_2_O rather than H_2_O was to maintain the high optical Q-factor, as H_2_O has strong optical absorption at 1550 nm. The optical Q-factor of the optical resonator in D_2_O was about 3 × 10^5^. Here, the Cr film was illuminated with 2.1 mJ, which generated PA signal with a peak pressure ~2 Pa in air as measured by the microphone. The air gap between the Cr film and the D_2_O filled chamber was ~1.4 mm. The distance between the top surface of D_2_O and the ring resonator was 2.0 mm. As seen in Fig. 7b, the ringing vibration is greatly dampened while demonstrating SNR of 22 (the averaging factor has been taken into account). The other subsequent peaks after initial peak were obtained too. However, due to small chamber size and possible multiple reflective boundaries, we were unable to identify the origin of each peak. The frequency response reveals broadened peak with center frequency = 840 kHz and FWHM = 200 kHz (Fig. 7c). Encapsulation of ring resonator reduces environmental noise^55^ and increases photoelastic effect^56^, which could be beneficial in enhancing SNR.

It should be noted that the bandwidth of the resonator is still narrow. This is due mainly to the excitation of specific mechanical resonance mode of the ring resonator (*e.g*., wineglass mode). When a mechanical mode is excited in resonators with a high mechanical Q-factor, a sharp peak is present at frequency response, thereby reducing the bandwidth of the resonator. In the absence of mechanical resonance modes, the acoustic detection bandwidth is limited only by the optical bandwidth of the resonator, which is about 200 MHz for our ring resonator with an optical Q-factor of 10^6^ at 1550 nm^24^.

## Discussion

In summary, we report the sensitive air-coupled detection of ultrasound wave using capillary-based optical ring resonators, with an NEP of 215 mPa/√Hz and 41 mPa/√Hz at 50 kHz and 800 kHz, respectively. We investigated the underlying theory and experimentally validated geometry-dependent pressure sensitivity. The practical detection limit of our ring resonator was comparable to commercially available products. Further experiments with PA signal revealed that our detector in air has a resonance frequency around 1.65 MHz, with an SNR of 10. We tested the feasibility of using encapsulated ring resonators in PA detection by immersing the ring resonator into D_2_O to broaden the frequency response. By the immersion, we observed a broadened frequency response (FWHM = 200 kHz) with SNR of 22.

In the future, to obtain broader frequency response, materials such as viscous low-refractive polymer could be used for the mechanical damping. Furthermore, the direction of the impinging acoustic pulse onto the resonator should be investigated so that mechanical resonance modes or surface acoustic waves will not be excited near the circumference where the optical mode is present. To increase the sensitivity of the capillary-based ring resonator, thinner walled capillaries (wall thickness ranging from sub-micron to ~2 μm)^40, 42, 56^ will be used, which can increase the sensitivity at least five-fold, while still maintaining excellent optical Q-factors. The performance of the ring resonator can be further improved by using a balanced detector (refer to Supplementary Information) to reduce common-mode noises^57^. In addition, various liquids can be flowed through the capillary, which exploits the larger photoelastic coefficients of liquids for enhanced sound wave detection sensitivities^56^.

Our work will lead to the development of compact and highly sensitive ultrasound detectors for air-coupled non-destructive ultrasound testing, PA imaging, and remote sensing. Furthermore, it will provide a model system for fundamental study of the mechanical modes in a high Q ring resonator. Finally, excitation of those mechanical modes using photoacoustic pulses may allow us to sensitively detect bio-analytes (such as proteins and cells) in the proximity of the ring resonator.

## Electronic supplementary material

Supplementary information
