# Supplementary material for: Air-coupled ultrasound detection using capillary-based optical ring resonators
Source: Sci Rep. 2017 Mar 1;7:109. doi: 10.1038/s41598-017-00134-7 (PMC5427941; doi:10.1038/s41598-017-00134-7)
Supplement: Supplementary file 1 — Supplementary information [file 41598_2017_134_MOESM1_ESM.pdf]

Air-coupled ultrasound detection using capillary-based optical ring resonators: Supplmentary Information

Kyu Hyun Kim[1,2], Wei Luo[2,3], Cheng Zhang[2], Chao Tian[1], L. Jay Guo[2], Xueding Wang[1], and Xudong Fan[1,*]

[1]Department of Biomedical Engineering, University of Michigan,1101 Beal Ave., Ann Arbor, MI 48109, USA
[2]Department of Electrical Engineering and Computer Science, University of Michigan, 1301 Beal Ave., Ann Arbor, MI 48109, USA
[3]School of Optical and Electrical Information, Huazhong University of Science and Technology, 1037 Luoyu Road, Hongshan District, 430074 Wuhan, Hubei, PR China
[*]Corresponding author: xsfan@umich.edu

Abstract

This document is supplementary document for to "Air-coupled ultrasound detection using capillary-based optical ring resonators." Additional information on calibration method, detecting using a balanced photodetector and mechanical resonance modes are discussed.

S1. Transducer calibration

In the experiment, an air coupled transducer (Japan Probe 0.9K14x20N-RX) was used to generate the ultrasound. There was an air space (approximately 2 cm) between the transducer and the resonator. It is necessary to estimate the acoustic pressure levels reaching the resonator under different external driving voltages. Usually a well-calibrated hydrophone can be employed to estimate the acoustic pressure in water. However, in the case of air, hydrophone cannot be used directly in air due to big acoustic impedance mismatch between air and the hydrophone surface. Furthermore, calibrated microphone at this frequency range is not readily available. For our current work, we calibrated the transducer in an indirect manner as described as follows.

A transducer can be treated as an ultrasound generator with pressure intensity (A_0) at given driving voltage, which is coupled to an outside environment through a multi-layered structure that has an equivalent acoustic impedance of Z_0 (see Fig. S1 for illustration). A_0 and Z_0 are intrinsic to the transducer and important in obtaining the pressure exiting the transducer at a given driving voltage. The equation governing the output pressure at the outer surface of transducer is given as:

$$B = \frac{2Z_m}{Z_0 + Z_m} A_0 \qquad \textbf{(1)}$$

where A_0 is the input pressure at the inner side of the transducer surface at a given driving voltage. Z_0 is the effective acoustic impedance of the transducer. B is the output pressure at the location very close to the transducer, but separated by a medium with an impedance of Z_m (see Fig. S1). If the output pressure can be measured inside two different media with known acoustic impedances, A_0 and Z_0 can be calculated from:

$$A_0 = \frac{B_2 B_1 (Z_2 - Z_1)}{2(B_1 Z_2 - B_2 Z_1)} \qquad \textbf{(2)}$$

$$Z_0 = \frac{(B_2 - B_1)Z_1 Z_2}{B_1 Z_2 - B_2 Z_1} \quad \textbf{(3)}$$

where subscript 1 and 2 stand for two different liquid media. Since Z_{air}, the acoustic impedance of air, is well-known, the output pressure of the transducer in air can be calculated using the following equation:

$$B = \frac{2Z_{air}}{Z_0 + Z_{air}} A_0 \quad \textbf{(4)}$$

Finally, the pressure at the resonator can be obtained by taking into account the attenuation of the pressure wave when it travels in air[1].

Figure S2a illustrates the experimental setup. A hydrophone (ONDA HNR-0500) was used to measure the pressure within a liquid medium. This is because due to the impedance mismatch between the hydrophone surface and air, the surface of the hydrophone would act as a reflecting surface if it were used in air.

To overcome such an issue, in the experimental setup in Fig. S2a, the hydrophone was immersed in water so that the unwanted reflection from the hydrophone could be minimized since the hydrophone is designed to match the impedance of water. A thin membrane chamber was used to separate water and the liquid medium. In our experiment, we used isopropyl alcohol and sodium silicate as two different liquid media with acoustic impedances of 0.919 MRayl (10^6 N-s/m^3) and 2.6035 MRayl, respectively. Ignoring ultrasound attenuation in liquids due to the short propagation distance, the pressure reading of the hydrophone, C, is related to the pressure at the output of the transducer, B_1 and B_2, by:

$$B_1 = \frac{Z_1 + Z_{water}}{2Z_{water}} C_1$$
$$B_2 = \frac{Z_2 + Z_{water}}{2Z_{water}} C_2. \quad \textbf{(5)}$$

By plugging Eq. (5) into Eqs. (2), (3), and (4), the output pressure the transducer can be deduced. Since the shape of the transducer head are rectangular and the geometry are comparable to the propagating distance in our experiment, the distance dependence of pressure decrease due to ultrasound beam divergence can be ignored. However, attenuation of the ultrasound in air due to absorption needs to be taken into account[1], which is about 104 dB/m for 800 kHz. Figure S2b is the calibration curve that relates the pressure (peak-to-peak) at the resonator in air to the input driving voltage (peak-to-peak).

S2. Pressure sensitivity enhancement using a balanced photodetector

Inherit noise in a laser source can lead to decreased sensitivity in optical resonator-based sensors. Here we confirm that by using a balanced photodetector to remove the laser noises and improve pressure sensitivity[2]. Balanced photodetectors have two optical inputs. One input collects the light from the laser source, while the other collects the signal of interest. At the output of the balanced photodetector, common-mode noises are removed.

In this experiment, the laser was split into two paths using a fiber coupler (Fig. S3a). One path sent the laser to the tapered fiber where ultrasound was detected using the resonator, while the other path sent laser to the reference channel of a balanced detector (New Focus 1617). The output of the tapered fiber, which carried the detected ultrasound signal, was again split into two paths. One path sent the ultrasound signal to the single input photodetector (New Focus 1611) and the other path sent the ultrasound signal to the signal channel of the balanced photodetector. Before ultrasound transducer was turned on, the difference between DC-intensities of reference and signal channels of the balanced detector was minimized using a fiber attenuator to ensure proper noise rejection. When ultrasound transducer was turned on, the difference between DC-intensities of reference and signal channels was continuously monitored. The radius of the resonator was 85 μm. The distance between the detector and the transducer was ~16 cm. Figs. S3b and S3c show the temporal response from each photodetector towards air-coupled ultrasound signal generated by a 50 kHz Tx (AirMar AR 50). The data was averaged 512 times. No filtering around 50 kHz was performed. The balanced photodetector had 37% higher SNR compared to single input photodetector.

S3. Mechanical resonance modes of a ring resonator

It is well known that a mechanical system can exhibit resonance behaviors at specific frequencies. Due to the annular shape and the homogeneity of the material, silica based ring resonators without damping layers are usually very good mechanical resonators with high mechanical Q-factors[3-6]. Different types of mechanical resonance modes exist for capillary based silica ring resonators with outer radius of ~50 - 100 μm and thickness of ~10 - 20 μm: (1) Radial mode (or breathing mode) around 10-100 MHz[4,7], (2) Surface acoustic modes around 100MHz - 11 GHz[5], and (3) Wineglass mode around 1 - 30 MHz[5,7].

In this work, the resonator had an outer radius of 80 μm and thickness of 11 μm. When the broadband acoustic wave generated from a chromium film reaches the resonator, it can excite different mechanical resonance modes of the resonator. The strongest mechanical resonance mode observed was around 1.65 MHz for this experiment. The finite element method calculation (COMSOL Multiphysics) reveals that there is a wineglass mode around 1.65 MHz as seen in Fig S4. The reason why wineglass mode dominates can be explained by the direction of the impinging acoustic wave, as the side-excitation of the resonator by the acoustic wave makes it easier to excite a wineglass mode.

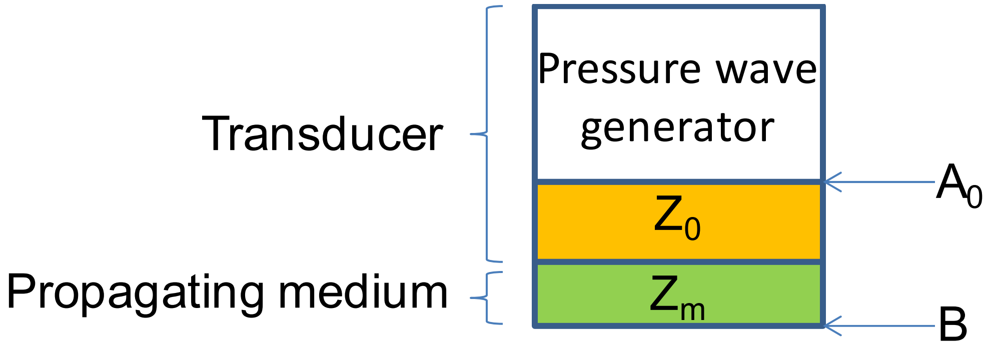

Figure S1. Illustration for the transducer model used in pressure estimation. Z_0 is the effective acoustic impedance of the transducer surface. Z_m is the acoustic impedance of propagating medium. A_0 is the pressure at the back of the transducer surface at a given driving voltage. B is the pressure in the propagating medium close to the transducer surface

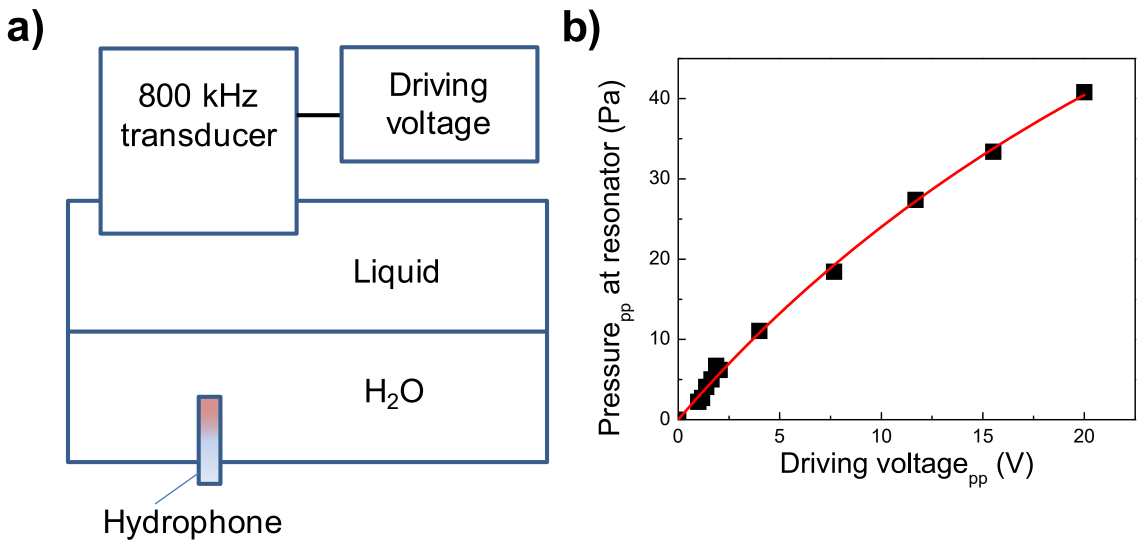

Figure S2. a) Illustration of the experimental setup for pressure calibration. The transducer was immersed in liquid with known acoustical impedance. Since the hydrophone was immersed in water, acoustic reflection from the hydrophone surface was minimized. A thin membrane chamber was used to separate water and liquid medium. An external driving voltage was used to drive the transducer. **b)** Pressure calibration curve. Peak-to-peak pressure values at the resonator in air as a function of the peak-to-peak value of driving voltage are given. Solid squares are experimental results. The red line is the curve fit using the Hill function, $y=V*x/(k+x)$, where V=128.71 and k=43.6.

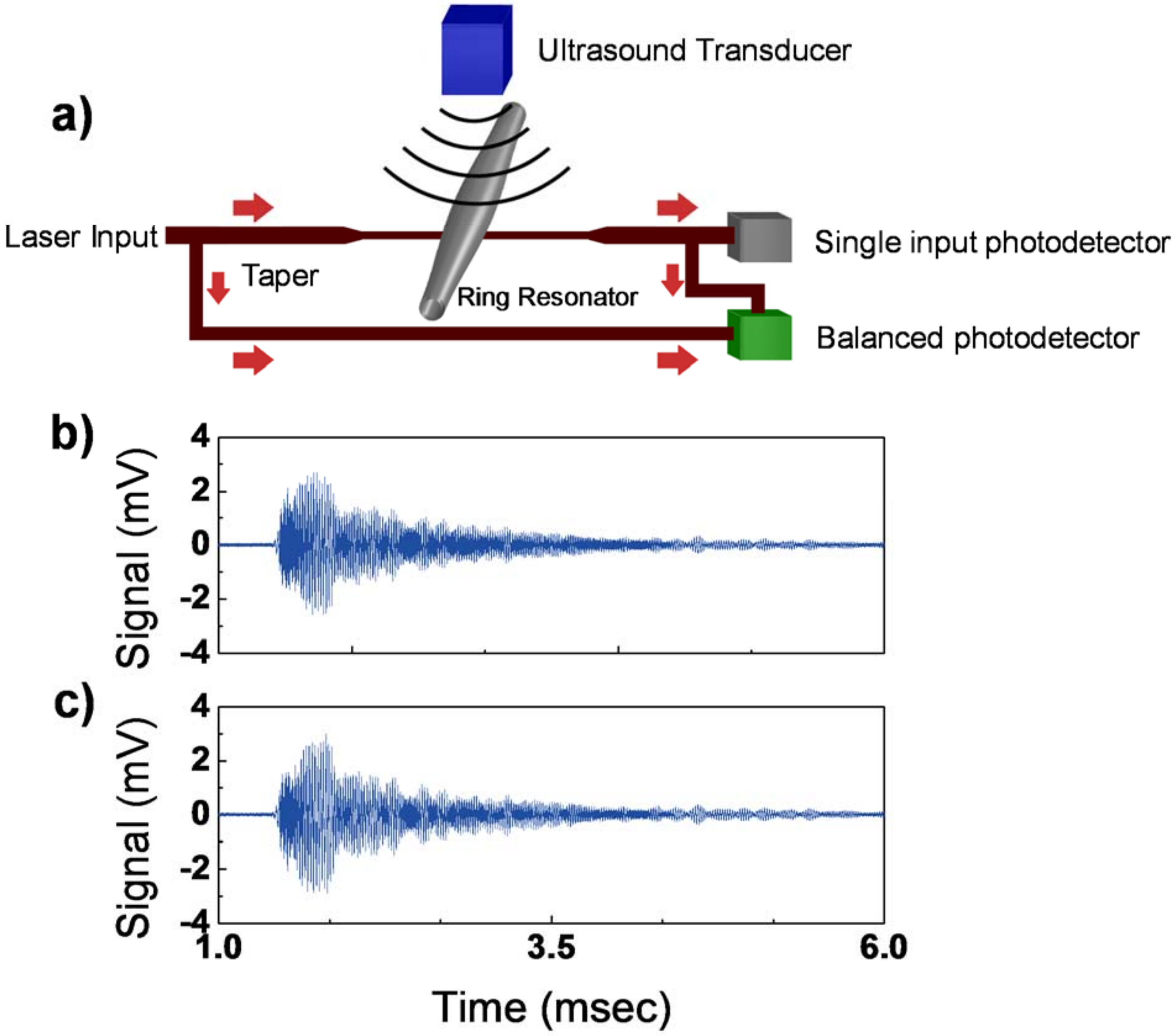

Figure S3. a) Schematic of the experiment. The intensity of the laser output to the balanced detector was adjusted so that it matches base level of the signal output from the taper. **b-c)** temporal responses of a ring resonator towards the 50 kHz air-coupled ultrasound. **b)** Temporal response recorded by a single input photodetector. Measured SNR was 6.5. **c)** Temporal response recorded by a balanced photodetector. Measured SNR was 8.8.

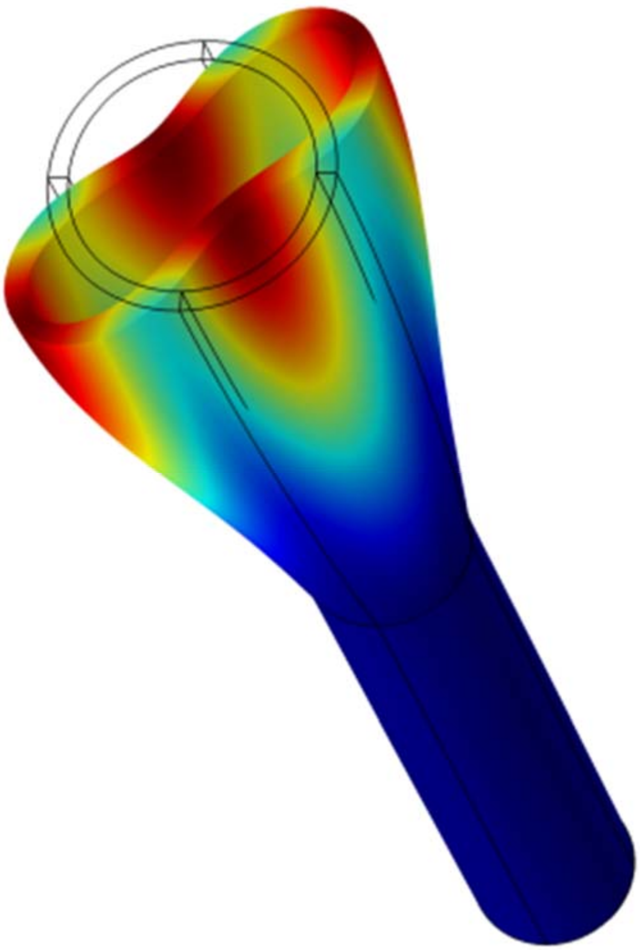

Figure S4. Calculated wineglass mode of the ring resonator using finite element method. The mechanical mode at 1.65 MHz is a wineglass mode of the resonator. Wireframe shows the ring resonator's geometry before the deformation. Color and deformation show the acoustic amplitude and displacement of the resonator.

References

1. ISO 9613 – 1. Acoustics. Calculation of the absorption of sound by the atmosphere. (2015).
2. Yalcin, A. *et al.* Optical sensing of biomolecules using microring resonators. *IEEE J. Sel. Topics Quantum Electron.* **12**, 148-155 (2006).
3. Carmon, T., Rokhsari, H., Yang, L., Kippenberg, T. J. & Vahala, K. J. Temporal Behavior of Radiation-Pressure-Induced Vibrations of an Optical Microcavity Phonon Mode. *Phys. Rev. Lett.* **94**, 223902 (2005).
4. Kim, K. H. *et al.* Cavity optomechanics on a microfluidic resonator with water and viscous liquids. *Light Sci. Appl.* **2**, e110 (2013).
5. Bahl, G. *et al.* Brillouin cavity optomechanics with microfluidic devices. *Nat. Commun.* **4**, 1994 (2013).
6. Tomes, M. & Carmon, T. Photonic micro-electromechanical systems vibrating at X-band (11-GHz) rates. *Phys. Rev. Lett.* **102**, 113601 (2009).
7. Zhu, K., Han, K., Carmon, T., Fan, X. & Bahl, G. Opto-acoustic sensing of fluids and bioparticles with optomechanofluidic resonators. *Eur. Phys. J. Spec. Top.* **223**, 1937-1947 (2014).
